# Current status of newborn hearing screening in low-income families in the southeastern region of Korea

**DOI:** 10.4178/epih.e2018044

**Published:** 2018-09-14

**Authors:** You Sun Chung, Su-Kyoung Park

**Affiliations:** 1 Departments of Otorhinolaryngology-Head and Neck Surgery, Gyengju Hospital, Dongguk University College of Medicine, Gyengju, Korea; 2 Department of Otorhinolaryngology-Head and Neck Surgery, Kangnam Sacred Heart Hospital, Hallym University College of Medicine, Seoul, Korea

**Keywords:** Newborn, Hearing loss, Hearing tests, Aural rehabilitation

## Abstract

**OBJECTIVES:**

The aim of this study was to analyze the current status and problems of hearing screening tests for newborns in low-income families in the southeastern Korea.

**METHODS:**

This study analyzed data from the Ministry of Health and Welfare’s project on the early detection of hearing loss in newborns in low-income families in the southeastern Korea (2011-2015).

**RESULTS:**

The referral rate was 1.33, 1.69, and 1.27% in Daegu, Gyeongbuk, and Ulsan, respectively. The confirmatory test rate was 36.09, 23.38, and 52.94% in Daegu, Gyeongbuk, and Ulsan, respectively. The incidence of hearing loss (adjusted) was 0.41, 0.62, and 0.41% in Daegu, Gyeongbuk, and Ulsan, respectively. After confirming hearing loss, newborns with hearing handicaps were mostly lost to follow-up, and rehabilitation methods, such as hearing aids or cochlear implants, were not used. The screening tests were performed within 1 month of birth, and the confirmatory tests were generally performed within 3 months of birth. However, more than 3 months passed before the confirmatory tests were performed in infants with risk factors for hearing loss in Gyeongbuk and Ulsan.

**CONCLUSIONS:**

Hearing screening tests were conducted in newborns from low-income families in southeastern Korea who received a coupon for free testing, but the newborns that were referred after the screening tests were not promptly linked to the hospitals where confirmatory tests were performed. Furthermore, hearing rehabilitation was not consistently performed after hearing loss was confirmed. To successful early hearing loss detection and intervention, a systematic tracking system of hearing loss children is needed.

## INTRODUCTION

Hearing loss is one of the most common diseases in newborns. Three to five out of every 1,000 newborns suffer from some form of congenital bilateral hearing loss above 40 decibels (dB), and 1-2 out of every 1,000 newborn babies have severe hearing loss [1-4]. Newborn babies who are hospitalized in an intensive care unit have been reported to have a hearing loss rate of 2-5% [5]. Hearing loss in newborns and infants is caused by genetic factors (40%); infections (31%), such as rubella and meningitis; causes related to birth (17%), such as premature birth, underweight, and complications; toxic drugs (4%); or other causes (8%) [6]. At birth, the peripheral organs of the auditory system are completely developed, but maturation of the auditory cortex is dependent on sound stimulation within 2-3 years of birth. After this period, the brain’s plasticity decreases, which can lead to limitations of language development, even with aural rehabilitation [7].

A prospective study was performed on 150 hearing-impaired newborns who began hearing rehabilitation within 2 months after the detection of hearing loss. The children were divided into 2 groups: (A) newborns with hearing loss detected before 6 months of life; and (B) newborns with hearing loss detected after 6 months. The children in group A had more advanced language development than those in group B. Therefore, for effective hearing development and rehabilitation, hearing loss should be diagnosed by at most 6 months after birth, and treatment for hearing impairment should be initiated as soon as possible [4].

Newborn hearing screening tests have been regularly conducted in developed countries since 1990 and were introduced in Korea in the 2000s. The type of hearing loss targeted by these tests is permanent unilateral or bilateral conductive or sensoryneural hearing loss, including auditory neuropathy, averaging 30-40 dB or more (over the frequencies 0.5, 1.0, 2.0, and 4.0 kHz) [1,8]. All newborns are recommended to undergo a hearing screening test using an automated auditory brainstem response test (AABR) or an automated otoacoustic emissions test (AOAE) within 1 month of birth. Newborns who do not show a clear response in1or both ears on the hearing screening test are required to undergo a confirmatory hearing test within 3 months after birth, and an early intervention should be implemented promptly after the detection of hearing loss, within 6 months after birth [1].

Approximately 50% of newborns and infants with hearing loss have at least 1 risk factor for hearing loss, with the remaining 50% having no risk factors. Currently, most developed countries conduct hearing screening tests on all newborns [1]. A universal newborn hearing screening test has not yet been implemented as a national project in Korea. However, the Ministry of Health and Welfare conducted a pilot project for the early detection of hearing loss of newborns in low-income families as part of a policy to encourage childbirth for 2 years from 2007 to 2008, and then has provided support for this project since 2009 [9]. Starting in the second half of 2018, health insurance will cover newborn hearing screening tests for the early diagnosis of congenital hearing loss according to the Ministry of Health and Welfare’s 2015 plan to strengthen health insurance coverage [10].

Before the implementation of health insurance coverage for hearing screening tests on all newborns in Korea, the current status of designated hospitals and each region regarding hearing screening tests should be examined and analyzed, and any problems detected should be corrected. Thus, an appropriate level of testing should be carried out irrespective of the region.

This study analyzed hearing screening data from newborns in low-income families in the southeastern region that received support from the Korean Ministry of Health and Welfare. The current status and problems of newborn hearing screening in these infants were assessed to obtain information relevant for the quality control of newborn hearing screening tests when universal hearing screening of all newborns is performed at the national level.

## MATERIALS AND METHODS

This study analyzed the southeastern region of Korea (Daegu, Gyeongbuk, and Ulsan) from 2011 to 2015, using the data set of 2the Ministry of Health and Welfare’s project on the early detection of hearing loss in newborns in low-income families in 17 cities and provinces. The distribution and status of the designated screening hospitals were analyzed from 2013 to 2015.

**Figure 1.**
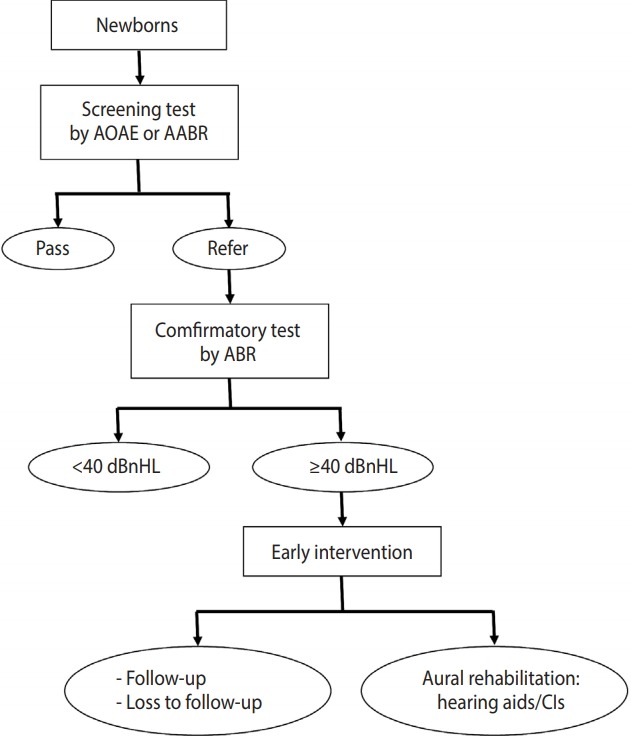
Flow chart of newborn hearing screening in Daegu, Gyeongbuk and Ulsan (2011-2015). AOAE, automated otoacoustic emission; AABR, automated auditory brainstem response; ABR, auditory brainstem response; dBnHL, decibel normal hearing level; CIs, cochlear implants.

The project was carried out as follows (Figure 1). The caregiver of the target babies applied for and received a free coupon for a newborn hearing screening test at a health center. Within 1 month after birth, a screening test was conducted at a designated screening hospital after the coupon was submitted. If either of the ears did not show a clear response during screening, a confirmatory hearing test at the otorhinolaryngology department was requested within 3 months after birth. Newborns with confirmed hearing loss above 40 dB normal hearing level (dBnHL) were recommended to receive aural rehabilitation, such as hearing aids or cochlear implants, within 6 months after birth [1]. The newborn hearing screening project support team of the Ministry of Health and Welfare established a comprehensive plan for the project and served as a consultation desk for inquiries from the national health centers, designated hospitals, and parents. The support team also collected the coupons that had been submitted to the health centers and the Ministry of Health and Welfare, and monitored the project in a comprehensive manner [9].

The following evaluation indices were examined. The screening rate was the percentage of newborn babies screened in a given region. The referral rate was the percentage of newborns who did not pass the hearing screen on either 1or both ears and were referred for a hearing confirmatory test among the newborns who were screened. This was preferably less than 4% of healthy newborns [1]. The confirmatory test rate was the percentage of referred newborns who received an auditory brainstem response (ABR) test as a confirmatory hearing test. More than 90% of newborns for whom a referral is indicated on the basis of the screening test should receive a confirmatory test for hearing loss within 3 months after birth [1]. The number of newborns with confirmed hearing loss was defined as the number of newborns with more than 40 dBnHL of hearing loss on the ABR test in either 1or both ears. The prevalence of hearing loss was the percentage of newborns with a hearing impairment above 40dBnHL among the newborns who were screened. Not all newborns who were referred received a confirmatory test. Therefore, the prevalence of hearing loss was corrected to the rate at which the hearing confirmatory test was performed. The prevalence of hearing loss by severity was calculated using each ear separately. A hearing loss of 40-55 dBnHL was classified as moderate hearing loss, 56-70 dBnHL as moderate to severe hearing loss, 71-90 dBnHL as severe hearing loss, and 91 dBnHL or higher as deaf or profound hearing loss. The early intervention rate was the percentage of newborns with confirmed hearing loss who received aural rehabilitation, such as hearing aids or cochlear implants, were lost to follow-up, or were followed up regularly. More than 95% of newborns should receive aural rehabilitation within 1 month after the confirmation of bilateral hearing loss [1]. The time from birth to screening and the time from birth to confirmation were recorded. The screening test should be performed within 1 month of birth and a confirmatory test should be performed within 3 months of birth [1]. Two technologies are used for hearing screening tests in newborns: AABR and AOAE. An ABR test was used to confirm hearing loss and to judge the hearing threshold objectively [2]. The types of hospitals designated for screening were divided into obstetrics and gynecology (OBGY) clinics; ear, nose, and throat (ENT) clinics at general hospitals; and private clinics. Areas with fewer hospitals delivering babies and expedition fertility rate were examined. Whether a newborn was at high risk for hearing loss was determined using the guidelines of the American Academy of Pediatrics, Joint Committee on Infant Hearing [1]. The statistical analysis was performed using SPSS version 20.0 (IBM Corp., Armonk, NY, USA), and the Mann-Whitney U test, Kruskal-Wallis test, and chi-square test were used to analyze the data. p-values less than 0.05 were considered to indicate statistical significance.

### Ethics statements

The study protocol was approved by the institutional review board (IRB) of Dongguk University Gyeongju Hospital (IRB no. 110757-201805-HR-07-02).

## RESULTS

### Data on newborn hearing screening

In 2015, 41 OBGY clinics, 10 ENT clinics of general hospitals, and 2 private clinics participated in this project in the southeastern region (Daegu, Gyeongbuk, and Ulsan). In the southeastern region from 2011 to 2015, 274,222 newborns were involved, 35,100 coupons were issued, and 33,108 newborns were screened. The screening rate was 12.07%, the referral rate was 1.50%, the confirmation rate was 31.85%, and the prevalence of hearing loss (after correction) was 0.48%. The average time from birth to screening was 4.65 days and the time from birth to confirmation was 72.21 days. Of the screening tests, 84.51% were performed by AABR and the expedition fertility rate was 13.56%. The screening rate was 9.96, 14.22, and 11.45% and the referral rate was 1.33, 1.69, and 1.27% in Daegu, Gyeongbuk, and Ulsan, respectively (p<0.05). The confirmatory test rate was 36.09, 23.38, and 52.94% in Daegu, Gyeongbuk, and Ulsan, respectively. Gyeongbuk showed a significantly lower confirmatory test rate than the other regions (p<0.05). The prevalence of hearing loss after correction was 0.41, 0.62, and 0.41% in Daegu, Gyeongbuk, and Ulsan, respectively (p>0.05). The severity of hearing loss was different in each region (p<0.05). A difference in to the types of early intervention in each region was observed. Loss to follow-up was the most common outcome in all 3 regions. Follow-up was more common in Gyeongbuk than in the other regions, but this tendency was not significant. The time from birth to screening was 3.27-6.11days and the time from birth to confirmation was 68.95-75.78 days in these areas. AABR was performed more than AOAE in all 3 regions. AABR was used significantly more in Daegu than in the other regions (p<0.05). The expedition fertility rate of Gyeongbuk was significantly higher than that of the other regions (p<0.05). Of the mothers who resided in Gyeongbuk in 2015 but delivered in another region, 81.46% delivered in Daegu (Table 1).

### Current status by type of screening institution

In 2015, 15 OBGY clinics, 5 ENT clinics at general hospitals, and 1 private clinic participated in the hearing screening project in Daegu; 19 OBGY clinics, 4 ENT clinics at general hospitals, and 1 private clinic in Gyeongbuk did so; and 7 OBGY clinics, 1 ENT clinic at a general hospital, and no private clinics did so in Ulsan.

The data were reviewed according to the type of institution designated for screening from 2013 to 2015 (Table 2). In each region, hearing screening was performed most often at OBGY clinics, followed by ENT clinics at general hospitals and private clinics (p<0.05). The referral rate was significantly higher for ENT clinics at general hospitals in Daegu and the private clinic in Gyeongbuk (p<0.05). More than half of the hospitals had a referral rate of less than 1%, but there was no significant difference among the 3 regions.

### Well babies versus newborns with high-risk factors for hearing loss

In the southeastern region from 2011 to 2015, 3.53% of applicants were high-risk newborns, as were 9.80% of newborns with hearing loss (Table 3). Furthermore, 0.14% of well babies had hearing loss, while high-risk newborns showed 0.43% of prevalence of hearing loss. The interval from birth to screening was 4.12 days in well babies and 16.66 days in high-risk newborns, and the interval between birth and confirmation was 68.84 days in well babies and 119.68 days in high-risk newborns. Newborns with high-risk factors accounted for 2.42, 2.95, and 6.69% of applicants born in Daegu, Gyeongbuk, and Ulsan, respectively. More high-risk newborns were found in Ulsan than in the other regions (p<0.05). The percentage of high-risk newborns among the newborns with hearing loss was 20.00% in Daegu, 0.00% in Gyeongbuk, and 13.33% in Ulsan (p>0.05). The prevalence of hearing loss in Daegu was significantly higher in high-risk newborns than in otherwise well babies. Well babies in each region were screened significantly faster than high-risk newborns, although all newborns, including high-risk newborns, were screened within 1 month of birth. The confirmatory test took longer for the high-risk newborns than the well babies in each region, but the difference was not significant. The hearing confirmatory test was not performed within 3 months of birth in the high-risk newborns in Gyeongbuk and Ulsan.

### Geographical distribution of the designated hospitals

Although some cities in Gyeongbuk had multiple screening hospitals, there were many cities and counties (guns) with 1 (Gyeongsan-gun, Sangju-gun, Yeongju-gun, and Uljin-gun) or no screening hospitals (Table 4). In Gyeongbuk, there were only 3 hospitals where confirmatory tests were performed, in Gyeongju, Gumi, and Andong. In Ulsan, there were no screening hospitals in Ulju-gun.

## DISCUSSION

Initially, this study comprehensively reviewed the status and problems of hearing screening tests of newborns in low-income families in the southeastern region of Korea.

More than 95% of all newborn babies should be screened in the first month of life [1]. Because this study was conducted on newborns in low-income families who were given a free coupon that was supported by the Ministry of Health and Welfare, the screening rate was approximately 9.96-14.22%. However, the screening rate of newborns issued with a free coupon in each region was more than 94.32%. The screening rate is expected to increase if insurance coverage for the hearing screening test is strengthened and if the test fee is lowered. Although the screening test was performed later in high-risk babies than in well babies, the hearing screening test of eligible newborns in all 3 regions was performed within 1month of birth. Hearing screening of high-risk newborns maybe delayed because they are more likely to receive examinations and treatment for medical problems. The referral rate is preferably less than 4% for healthy newborns [1]. The referral rates of Daegu, Gyeongbuk, and Ulsan were 1.33, 1.69, and 1.27%, respectively, which appeared appropriate (Table 1). However, in an analysis according to the range of referral rates from 2013 to 2015, hospitals with a referral rate of less than 1% accounted for 58.46, 71.01, and 61.54% of hospitals in Daegu, Gyeongbuk, and Ulsan, respectively. Screening tests must be repeated at most twice in each ear at the same time [11]. Repeating the test 3 or more times makes it possible to miss a hearing-impaired newborn due to the possibility of accidentally passing, resulting in a lower referral rate [12]. Therefore, if the referral rate is too low, it is necessary to check how many times the screening test was carried out at the same time. The referral rate, particularly of OBGY clinics, which accounted for more than half of the screening institutions, was lower than that of general hospitals in all 3 regions and that of the private clinic in Gyeongbuk. The reason for the low referral rate of OBGY clinics in these 3 regions needs to be examined for future quality control (Table 2).

More than 90% of the newborns referred by screening should receive a confirmatory test within 3 months of birth [1]. The confirmation rate was 36.09, 23.38, and 52.94% in Daegu, Gyeongbuk, and Ulsan, respectively (Table 1). The babies who were referred after screening were not promptly linked to the confirmatory test. The confirmatory test in all 3 regions appeared to have been conducted within 3 months after birth, but when the newborns were divided into well babies and high-risk newborns, it was found that the confirmatory tests of high-risk newborns in Gyeongbuk and Ulsan were conducted after the age of 3 months, indicating a need for further management (Table 3).

Aural rehabilitation, such as hearing aids, after confirmed hearing loss should be started within 6 months of birth [1]. None of the newborns with hearing loss in the 3 regions received hearing aids or cochlear implants. Caregivers might not be aware of the need for an early intervention for hearing development, and early interventions might be difficult because the cost of hearing aids or cochlear implant surgery is a burden for low-income families. To increase the early intervention rate, parents must be educated about the importance of early interventions for hearing development. Moreover, if newborns with a hearing impairment meet the criteria for disability, ENT doctors should help them register as disabled, allowing them to receive government subsidies for hearing aids.

In this study, 4-6 of every 1,000 newborns from low-income families had hearing loss, which was similar to the rates reported in other studies [1-4]. High-risk newborns have been reported to have a higher prevalence of hearing loss than well babies [5]. In this study, high-risk newborns had an approximately 3 times higher prevalence of hearing loss (before correction) than otherwise well babies in the southeastern region of Korea. Interestingly, the prevalence of hearing loss in Gyeongbuk was higher in otherwise well babies (Table 3). This may have been due to the small number of target newborns in low-income families or the low confirmatory test rate in Gyeongbuk.

Screening by ABR was significantly more common than screening by AOAE. AABR was used much more often in Daegu than in the other regions (Table 1). AABR appears to have been used widely because it has a low referral rate and can identify more causes of hearing loss. AABR is a test that checks for cochlear abnormalities, auditory nerve lesions, and brainstem lesions, with a referral rate of approximately 4%. AOAE is a test to check for abnormalities of the cochlea, with a referral rate of approximately 7-8% [2]. Therefore, high-risk newborns should be tested with AABR because of the high likelihood of auditory neuropathy [1,2].

The problems regarding newborn hearing screening in each region were also reviewed. Considering the current situation in Daegu, the referral rate of OBGY clinics, which accounted for the largest number of primary screening institutions, was very low (0.72%). The number of tests performed on each ear at a time should be checked, especially at OBGY clinics in Daegu. Gyeongbuk had a high expedition fertility rate and did not have a constant distribution of screening institutions; there were no screening hospitals in 2 cities (Mungyeong and Yeongcheon) and 12 counties (Goryeong, Gunwi, Bonghwa, Seongju, Yecheon, Uiseong, Yeongdeok, Yeongyang, Ulleung, Cheongdo, Cheongsong, and Chilgok). No hospitals offering confirmatory tests were in the vicinity of Uljin, Yeongyang, and Yeongdeok-gun in the north-eastern part of Gyeongbuk (Table 4). Gyeongbuk should equip hospitals suitably in areas where hearing test facilities are insufficient, because easy access to hospitals can promote the proper timing of hearing screening tests. Gyeongbuk had a confirmatory test rate of 23.38%, which was lower than that of the other regions (p<0.05). The prevalence of hearing loss in Gyeongbuk was higher than in the other regions, but the difference was not significant. Therefore, the prevalence of hearing loss in Gyeongbuk may change if the confirmatory test rate increases. To increase the confirmatory test rate in Gyeongbuk, the screeners or responsible doctors should be aware of the importance of newborn hearing screening, and particularly of hearing loss in high-risk patients. They should educate caregivers about the significance of newborn hearing screening and encourage referred newborns to receive a confirmatory test.

In Ulsan, 6.69% of the newborns who were screened were at high risk, which was significantly higher than the corresponding rate in Daegu or Gyeongbuk. Fortunately, the confirmatory test rate was 52.94%, which showed that the linkage of newborns requiring a referral to the confirmatory test was better in Ulsan than in the other 2 regions, and the probability of missing a hearing-impaired high-risk newborn was somewhat lower. Efforts should be made to determine why there were more newborns with high-risk factors in Ulsan.

This study had some limitations. The results of this analysis alone may not be consistent with the results of hearing screening tests of all newborns in those areas in the future because this study analyzed data from the Health and Welfare Ministry’s project that provided support for the early diagnosis of newborn hearing loss in low-income families (2011-2015). For example, in low-income families, it is more likely that the mother and/or newborn will have poor health or nutritional status or be exposed to an environment harmful for hearing or to sources of infection. Additionally, purchasing hearing aids imposes an economic burden on low-income families. Therefore, the prevalence of hearing loss and the percentage of newborns at a high risk of hearing loss might be higher in the low-income families included in this study than in the overall population, and the early intervention rate after confirming hearing loss might be lower.

In conclusion, newborn hearing screening in low-income families in Daegu, Gyeongbuk, and Ulsan was implemented to some extent. However, most newborns who received a referral did not undergo a hearing confirmatory test. In addition, even if hearing loss was diagnosed, early interventions such as hearing aids or cochlear implants were not provided, meaning that newborns with hearing loss did not receive the appropriate help for hearing development. In particular, in newborns at high risk for hearing loss, a considerable time passed from birth to the confirmatory test. Therefore, quality control of testing in high-risk newborns is necessary.

## Figures and Tables

**Table 1.** Data of newborn hearing screening in Daegu, Gyeongbuk, and Ulsan (2011-2015)

	Southeastern region	Daegu	Gyeongbuk	Ulsan	p-value
Newborns	274,222	100,431	115,503	58,288	
Screening tests	33,108	10,003	16,429	6,676	
Screening rate (%)	12.07	9.96	14.22	11.45	<0.001^1^
Referral rate (%)	1.50	1.33	1.69	1.27	0.01^1^
Confirmatory test rate (%)	31.85	36.09	23.38	52.94	<0.001^1^
Newborns with hearing loss	51	15	21	15	
Prevalence of hearing loss (after correction, %)	0.48 ± 0.31	0.41 ± 0.25	0.62 ±0.43	0.41 ± 0.21	0.78^2^
Severity of hearing loss (dBnHL)					0.04^1^
Moderate (40-55)	32	5	17	10	
Moderate to severe (56-70)	17	9	2	6	
Severe (71-90)	3	1	1	1	
Profound (over 91)	15	2	9	4	
Early intervention rate (%) among newborns with bilateral or unilateral hearing loss					
Lost to follow-up	88.03 (n=46)	93.33 (n=14)	85.71 (n=18)	93.33 (n=14)	0.67^1^
Hearing aids	NA	NA	NA	NA	
Cochlear implants	NA	NA	NA	NA	
Follow-up	11.97 (n=5)	6.67 (n=1)	14.29 (n=3)	6.67 (n=1)	0.67^1^
Time from birth to screening (d)	4.65 ± 1.27	3.27 ± 0.32	4.58 ± 0.60	6.11 ± 0.38	0.002^2^
Time from birth to confirmation (d)	72.21 ± 13.10	75.78 ± 10.84	68.95 ± 8.04	71.91 ± 19.72	0.41^2^
AABR (%)	84.51	96.22	76.48	86.74	<0.001^1^
Expedition fertility rate (%)	13.56	1.72	23.98	5.66	<0.001^1^

dBnHL, decibels normal hearing level; AABR, automated auditory brainstem response; NA, not available.

1 By the chi-square test for comparisons between data in Daegu, Gyeongbuk, or Ulsan from 2011 to 2015.

2 By the Kruskal-Wallis test.

**Table 2.** Data by the type of designated institution for newborn hearing screening (2013-2015)

	Southeastern region	Daegu	Gyeongbuk	Ulsan	p-value^1^
Screening rate					<0.001
OBGY clinics	85.30	88.44	81.89	88.83	
ENT of general hospitals	13.85	10.97	16.77	11.17	
Private clinics	0.84	0.59	1.35	0.00	
Referral rate					<0.001
OBGY clinics	0.91	0.72	0.96	1.08	
ENT of general hospitals	3.96	5.40	3.96	1.79	
Private clinics	4.23	0.00	5.41	0.00	
Analysis of hospitals by the range of the referral rate					0.41
<1	64.38	58.46	71.01	61.54	
1-4	21.88	27.69	14.49	26.92	
≥ 4	13.75	13.85	14.49	11.54	

Values are presented as %. OBGY, obstetrics and gynecology; ENT, ear, nose, and throat.

1 By the chi-square test.

**Table 3.** Data of newborn hearing screening in well babies versus newborns with high-risk factors of hearing loss (2011-2015)

	Southeastern region	Daegu	Gyeongbuk	Ulsan	p-value
High-risk newborns among applicants (%)	3.53	2.42	2.95	6.69	<0.001^1^
High-risk newborns among newborns with hearing loss (%)	9.80	20.00	0.00	13.33	0.12^1^
Prevalence of hearing loss (%)					
In well babies	0.14	0.12	0.13	0.21	0.31^1^
In high-risk newborns	0.43	1.24	0.00	0.45	0.05^1^
p-value^1^	0.07	<0.001	0.99	0.53	
Time from birth to screening (d)					
In well babies	4.12±0.31	3.02±0.34	4.16±0.55	5.14±0.28	0.002^2^
In high-risk newborns	16.66±2.18	13.98±3.73	18.20±2.74	17.80±3.05	0.11^2^
p-value^3^	0.009	0.009	0.009	0.009	
Time from birth to confirmation (d)					
In well babies	68.84±4.31	74.56±12.10	62.36±16.22	69.54±15.08	0.46^2^
In high-risk newborns	119.68±34.24	82.40±7.53	144.25±10.25	141.80±102.11	0.45^2^
p-value^3^	0.009	0.30	0.05	0.44	

1 By the chi-square test.

2 By the Kruskal-Wallis test.

3 By the Mann–Whitney U test.

**Table 4.** Geographical distribution of hospitals designated for hearing tests in Daegu, Gyeongbuk, and Ulsan (2016)

Districts (total, n)	Daegu district, counties (8)	Gyeongbuk		Ulsan districts, counties (5)
		Cities (10)	Counties (13)	
Districts without a screening hospital		Mungyeong	Goryeong-gun, Gunwi-gun	Ulju-gun
		Yeongcheon	Bonghwa-gun, Seongju-gun, Yecheon-gun	
			Uiseong-gun, Yeongdeok-gun, Yeongyang-gun	
			Ulleung-gun, Cheongdo-gun	
			Cheongsong-gun, Chilgok-gun	
Districts without a confirmatory test hospital	Seo-gu, Buk-gu, Suseong-gu, Dalseo-gu, Dalseong-gun	Gyeongsan, Gimcheon	All 13 counties	Buk-gu, Ulju-gun
		Sangju, Pohang		
		Yeongju, Mungyeong, Yeongcheon		
